# Influence of femoral bowing on stress distribution of the proximal femur: a three-dimensional finite element analysis

**DOI:** 10.1186/s13018-023-03559-1

**Published:** 2023-02-01

**Authors:** Nobuhiro Kaku, Tsuguaki Hosoyama, Yutaro Shibuta, Makoto Kimura, Hiroshi Tsumura

**Affiliations:** Department of Orthopaedic Surgery, Faculty of Medicine, Oita University, 1-1 Idaigaoka Hazama-Machi, Yufu City, Oita 879-5593 Japan

**Keywords:** Femoral bowing, Stress distribution, Proximal femur, Finite element analysis

## Abstract

**Background:**

Whether femoral bowing or its direction has a mechanical effect on the proximal femur is unclear. This study aimed to define the changes in stress distribution in the proximal femur associated with femoral bowing using finite element analysis.

**Methods:**

We created four femoral models: original, entire lateral bowing, entire anterior bowing, and the middle of both (50% anterolateral bowing) from computed tomography data of women with standard bowing. Each model’s stress distribution was compared by two-layering the stress distribution under loading conditions during walking. We also evaluated displacement vectors.

**Results:**

In all directions of femoral bowing, the stress increased in the femoral neck and the femoral trochanter in the 50% anterolateral bowing. The direction of deformation of the vector for the femoral head increased anteroinferiorly in the 50% anterolateral bowing.

**Conclusions:**

This study showed that the stress distribution at the proximal femur shifted laterally. The high-stress area increased at the femoral neck or trochanter due to increasing femoral bowing. Femoral bowing also increases the anteroinferior vector in the femoral head. This study provides valuable insights into the mechanism of proximal femoral fractures in older adults.

**Supplementary Information:**

The online version contains supplementary material available at 10.1186/s13018-023-03559-1.

## Background

In many high- and middle-income countries, aging populations and low fertility have resulted in a high proportion of older adults [1]. Osteoporosis-based femoral neck and trochanteric fracture incidence rates are expected to increase in the future [2] and significantly impact life and daily activities [3]. Therefore, it is essential to elucidate the mechanism of occurrence and prevent its occurrence. In a Japanese epidemiological survey, the incidence of proximal femur fractures per 100,000 population was higher in women than in men [4]. In addition, other epidemiological studies reported that femoral neck fractures were more common in patients below 75 years of age, and trochanteric fractures were more common in those over 75 years [5], suggesting that the site of the fracture varies with age. Other factors, as well as osteoporosis, may be related to the cause of differences in fracture rates and sites by sex and age group.

Age-related deterioration of the locomotor system leads to changes in posture or organ geometry, osteoporosis, and sarcopenia. In addition, it has been found that pelvis tilt changes with age [6, 7]. As shown in previous studies, stress analysis using the finite element (FE) method has verified that stress on the articular surface of the hip joint increases when the pelvis is tilted posteriorly in the sagittal plane or the opposite hip is inclined distally in the coronal plane [8, 9].

The femur is the longest bone in the human body and has two axes, the femoral diaphysis, and the neck, which comprise the hip and knee joints. Long tubular bones such as the femur and tibia have been reported to change shape over time [10, 11]. The effect of changes in overall femoral geometry on stress distribution in the proximal and distal femur has, however, been unclear.

Femoral bowing was first described by Stewart [12]. Although the direction of femoral bowing is usually anterolaterally convex [11], the degree and direction vary among ethnic groups [13]. Moreover, even within the same ethnic group, there are also suggested associations with age, sex, shorter stature, bone density, bone quality, and quantity [14, 15]. In addition, sex and individual differences have been reported in the degree and direction of femoral bowing [11]. Tagomori H et al. showed that femoral curvature varies with age in Japanese individuals with three-dimensional evaluation using computed tomography (CT) data and that increase in femoral curvature led to position abnormality, such as a higher top of the greater trochanter [11].

Conversely, there have been a few reports on the effect of femoral bowing on the development and progression of the disease [16, 17]. Oh et al. referred to the association between femoral bowing and atypical fractures occurring in the middle of the femur [16]. Matsumoto et al. stated that age-related femoral bowing contributes to the development of medial-type knee osteoarthritis (OA), which is considered common in the Japanese population [17]. As in these reports, it can be inferred that differences in femoral bowing progression directly or indirectly affect the knee joint, cause trauma, and lead to the development of degenerative hip joint diseases or decreased muscle strength in the lower limb. However, there are no reports on the mechanical effects of femoral bowing on the proximal femur.

It is unclear whether severe femoral bowing changes the stress distribution in the proximal femur under load, or if it depends on its direction*.* By observing these basic phenomena, we believe it is possible to examine the mechanism of disease development. The FE method has recently been widely used in various fields to analyze stress distribution. This study aimed to investigate the effect of femoral bowing on stress distribution in the proximal femur in three dimensions using the FE method.

## Methods

The study was approved by the Ethics Committee of Oita University (approval number 1052; approval date, July 22, 2016) and was performed as per the ethical standards of the 1964 Declaration of Helsinki and its later amendments. The patients provided written informed consent for this study preoperatively.

**Fig. 1:**
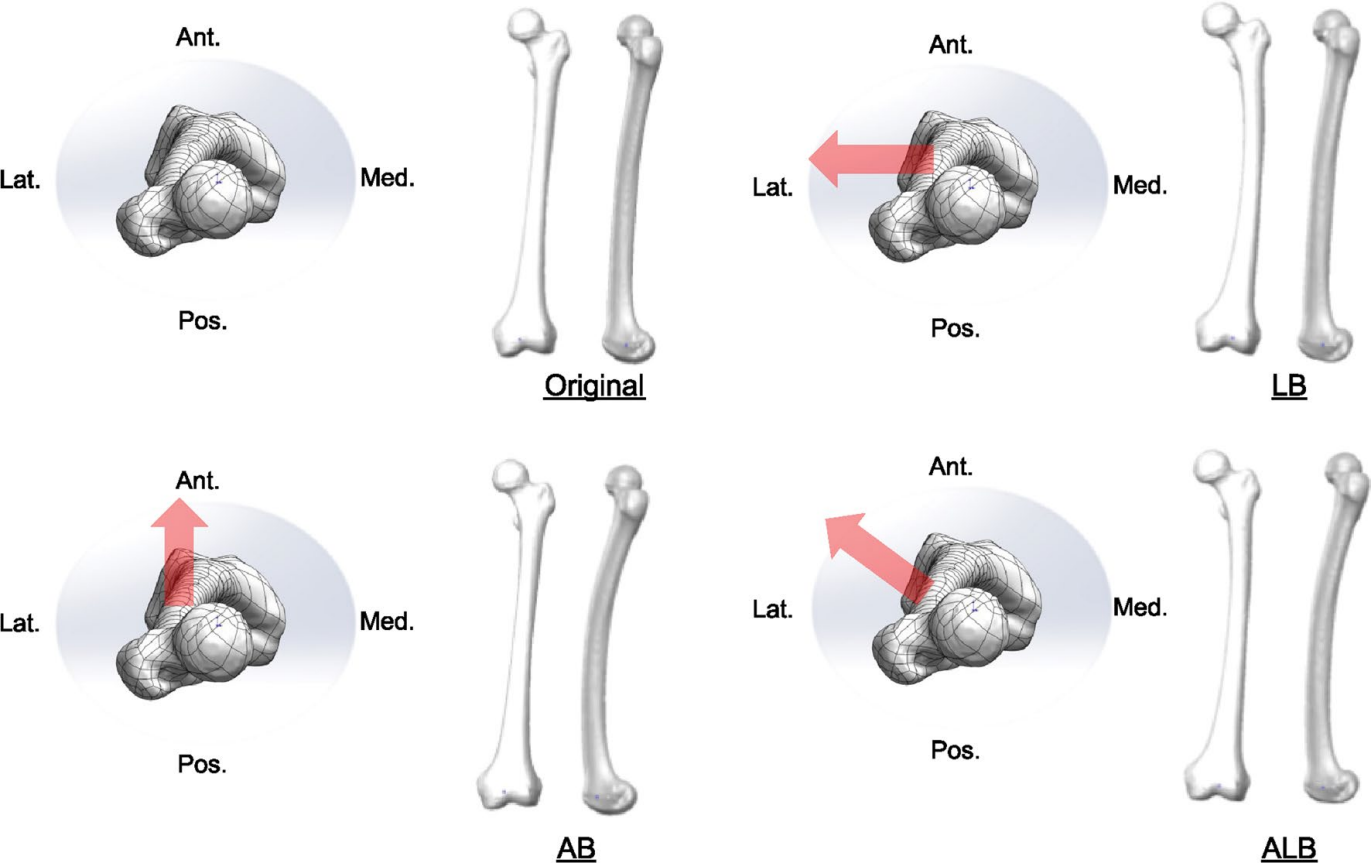
Designed femoral bowing model: The femur of an original was subjected to bowing. The bowing direction was defined as 100% lateral bowing in the coronal plane, 100% anterior bowing in the sagittal plane, and 50% anterolateral bowing

Forty individuals who underwent anterior cruciate ligament reconstruction or operative treatment for trauma in our hospital between January 2017 and May 2018 and had postoperative CT imaging of the total femur were included. CT images of the iliac wing to the knee joint were acquired using a helical CT scanner (Aquilion CX; Toshiba Medical Systems Corporation, Tokyo, Japan) with a 1-mm slice thickness. Femoral curvatures in the sagittal and coronal planes were measured individually, as in our previous study [11]. One patient with the least bowing was selected as the original model based on the results of a femoral curvature survey of all patients. From the CT data of the right side of a 17-year-old woman with the least bowing, a model with original and increased bowing was created. The length of the patient’s functional axis was 361.7 mm, and the three-dimensional femoral bowing obtained by acquiring three points of the medullary approximate circle center at the proximal, middle, and distal parts of the diaphysis [11] was R = 1061.2. A three-dimensional bony femur model was created from the CT image data of the selected subject using the three-dimensional medical image processing software Mimics (Materialise Inc. Leuven. Belgium). The data were used in their original form from the actual patient model. For the bowing model, the three-dimensional bone model was imported into the 3D CAD software SolidWorks (Dassault Systemes Inc., Massachusetts, USA). The flex function was used to give the original femur a bowing shape, and a femoral coordinate system was constructed for each model [18]. In the coronal plane, lateral bowing (LB) was 100%; in the sagittal plane, anterior bowing (AB) was 100%; and in the intermediate direction (the anterolateral direction), anterior and lateral bowing (ALB) were both 50% (Fig. 1). After constructing the femoral coordinate system, the inflection point of the bowing was located in the middle of the origin and the lesser trochanter. We set the degree of bowing to be equal to or greater than that of the patient with the most bowed femur among older adult patients with proximal femur fractures who underwent CT during the same period. The proximal femur curvature in each model was R = 678.1 for LB, R = 536.2 for AB, and R = 567.7 for ALB. Using the original and bowing femoral models, FE models were created and analyzed.

The CAE software Abaqus (Dassault Systèmes Simulia Corp., Johnston, Rhode Island, United States) was used for the creation and analysis. The anteversion angle of the original femur was 14.9°, and that of the bowing femur was LB: 14.53°, AB: 16.11°, and ALB: 16.84°. The femur model consisted of 4-node tetrahedral elements with an element size of approximately 2.0 mm. The number of elements in each model was 501.910 for the original, 520.163 for LB, 500.544 for 100% AB, and 511.152 for ALB. The material properties of cortical and cancellous bone are listed in Table 1.

**Table 1:** Material properties (linear elastic materials) used in the finite element simulations

	Young’s modulus (GPa)	Poisson’s ratio
Cortical bone	15.0	0.28
Cancellous bone	1.0	0.3

**Fig. 2:**
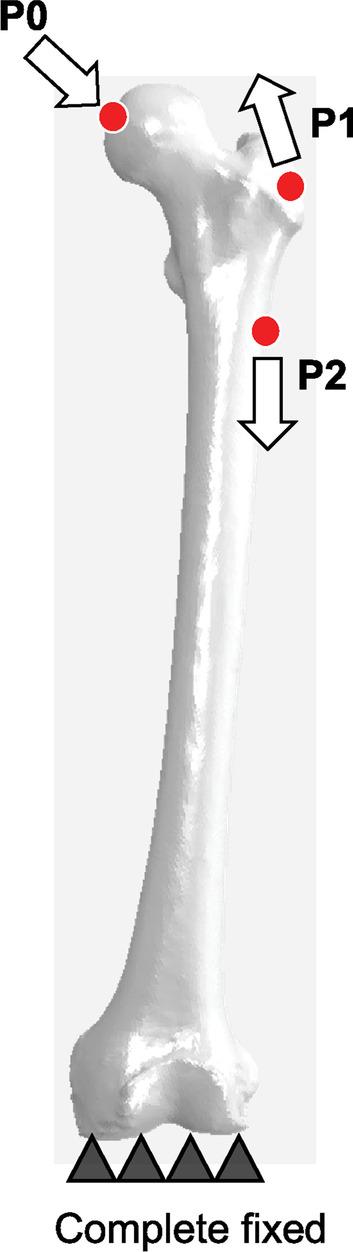
Load condition: Bergmann's loading condition for walking was used. The maximum load (joint reaction force) during walking was applied (P0), and the effects of the surrounding soft tissues were also considered (P1, P2) to simulate the stress transfer during walking

In the FE analysis, the femur was analyzed using a two-layered cortical and cancellous bone model (Table 1). The cortical bone was defined by shell elements, with a uniform thickness of 1 mm, and the distal femur was fully constrained. The influence of the surrounding soft tissue was also considered based on the report by Heller et al. [19] (Fig. 2). The condition of load constraint on the femur was assumed to be that of walking, and the maximum load (joint reaction force) during walking was applied to the femoral head in the femur reference coordinate system. We also calculated the load values from P0 to P2 based on the weight of the specimen and entered values at each point (Table 2). After analysis, a threshold value was defined as the value at which the high- and low-stress regions were both 50% of the original Mean Mieses stress distribution for the femur model. A binary stress map was obtained for each specimen based on the defined original threshold value.

**Table 2:** Load acting on the femur and stem during gait

	*X*	*Y*	*Z*
P0	− 270.1 N	− 164.0 N	− 1146.3 N
P1	32.4 N	7.6 N	40.4 N
P2	− 0.5 N	9.3 N	− 46.5 N

The three action points (P) of the attachments or wrapping points of the muscles are labeled as P0, P1, and P2

**Fig. 3:**
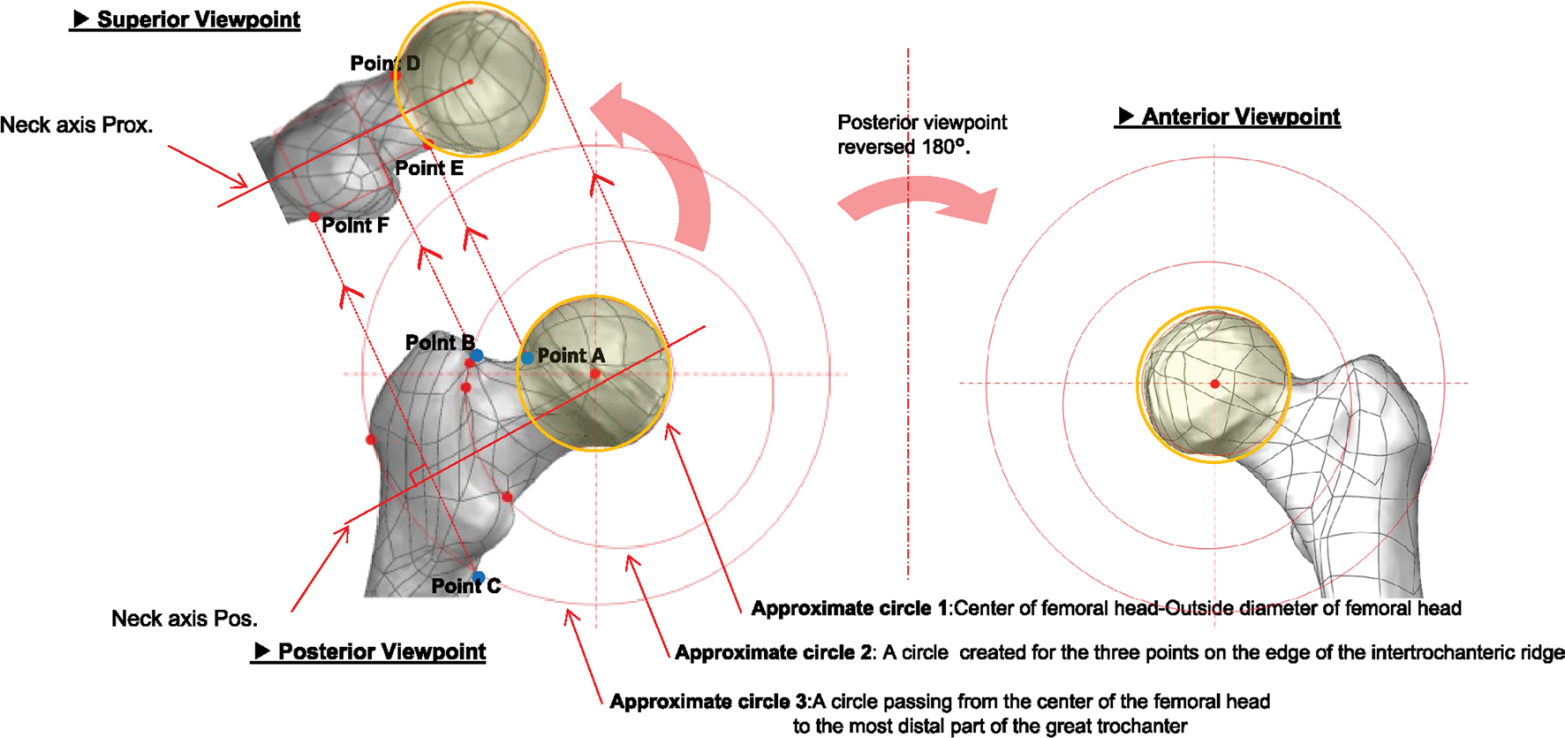
Region setting for stress measurement: A circle was drawn around the femoral head in the superior field of view, and the neck was defined from the outline of the circle of the femoral head to the intertrochanteric ridge, and the trochanter region was defined from the intertrochanteric ridge to the outer edge of the greater trochanter

To further facilitate the comparison of the calculated stress changes in this report, six regions of interest were set up at the neck and trochanter in three viewpoints: posterior, anterior, and superior (Fig. 3).

First, an approximate sphere 1 was created at the femoral head on the model. Then, from a posterior viewpoint, an approximate circle 2 was created for the three points on the edge of the intertrochanteric ridge. From the same perspective, circle 3 was created around the center of the femoral head, passing through the most lateral part of the greater trochanter. The region of interest of the neck in the posterior viewpoint is defined as the neck region divided by approximate sphere 1 and circle 2 on the femur model. Similarly, the region of interest of the trochanter was defined as the trochanter region divided by approximate circles 2 and 3. The regions of interest for the neck and trochanter region in the anterior viewpoint were defined similarly by applying the regions of interest mentioned above for the neck and trochanter region set in the posterior viewpoint directly to the anterior viewpoint.

Subsequently, the model was projected perpendicularly to the neck axis posterior, extracted from the backward viewpoint. This was defined as the superior viewpoint. The proximal intersection of the approximate sphere 1 and the model contour line of the femur was defined as point A. The proximal intersection of approximate circle 2 and the model contour line of the femur was designated as point B. Circle 3 and the model contour line of the femur were defined as point B. The distal intersection of circle 3 and the model contour line of the femur was designated as point C. In addition, from points A, B, and C, an extension line is drawn perpendicular to the posterior neck axis (Neck axis Pos) toward the projected, superior viewpoint. At the superior viewpoint, the intersection points of the extension line from point A and the approximate sphere A were designated as points D and E, respectively.

Similarly, the intersection of the extension line from point C and the rear side of the femur model contour line was designated as point F. Finally, an extension parallel to the proximal neck axis (Neck axis Prox) was drawn from points D, E, and F extracted from the superior viewpoint. In the superior viewpoint, the region of interest in the neck was defined by the extensions drawn from points A and B in the posterior viewpoint toward the superior viewpoint, as well as by the extensions drawn from points D and E in the superior viewpoint. Similarly, the region of interest on the trochanter in the superior viewpoint was defined by the extensions drawn from points B and C in the posterior viewpoint toward the superior viewpoint and the extensions drawn from points D and F in the superior viewpoint.

This study focused on the six regions of interest for the femoral neck and trochanter in three dimensions from the posterior, anterior, and superior views. We then compared the LB, AB, and ALB models with the original model before the shape change. The effect of the deformity on the stress distribution in the proximal femur, particularly in the neck and trochanter, was evaluated with a binarized stress map. We also obtained rotational displacement vectors of the femur before and after FE analysis. To facilitate comparison of the results among the specimens, the displacement vectors were displayed at the same scale.

We compared four groups using the Kruskal–Wallis test and two using the Steel–Dwass method to compare the stresses of all elements present in each region of interest in the four models. The comparison was performed using EZR (Saitama Medical Center, Jichi Medical University, Japan), a GUI of R (The R Foundation for Statistical Computing, Vienna, Austria, version 3.6.2) [20]. More precisely, it is an improved version of R commander (version 2.6-2), designed to incorporate statistical methods. After confirming significant differences between groups using the Kruskal–Wallis test, *p* values were calculated by multiple comparisons using the Steel–Dwass method. A *p* value of ≤ 0.05 was considered significant.

## Results

The thresholds in each region of interest were 9.8 MPa at the neck of the posterior viewpoint and 13.40 MPa at its trochanter, 11.20 MPa at the neck of the anterior viewpoint, 9.00 MPa at its torsion, 7.60 MPa at the neck of the superior viewpoint, and 7.20 MPa at its trochanter.

In the posterior viewpoint, stress at the neck increased in all directions of femoral bowing, with a maximum at LB. The stress at the trochanter decreased at 100% LB and AB and increased slightly at ALB. In the posterior viewpoint, stress increased more at the neck than at the trochanter.

**Fig. 4:**
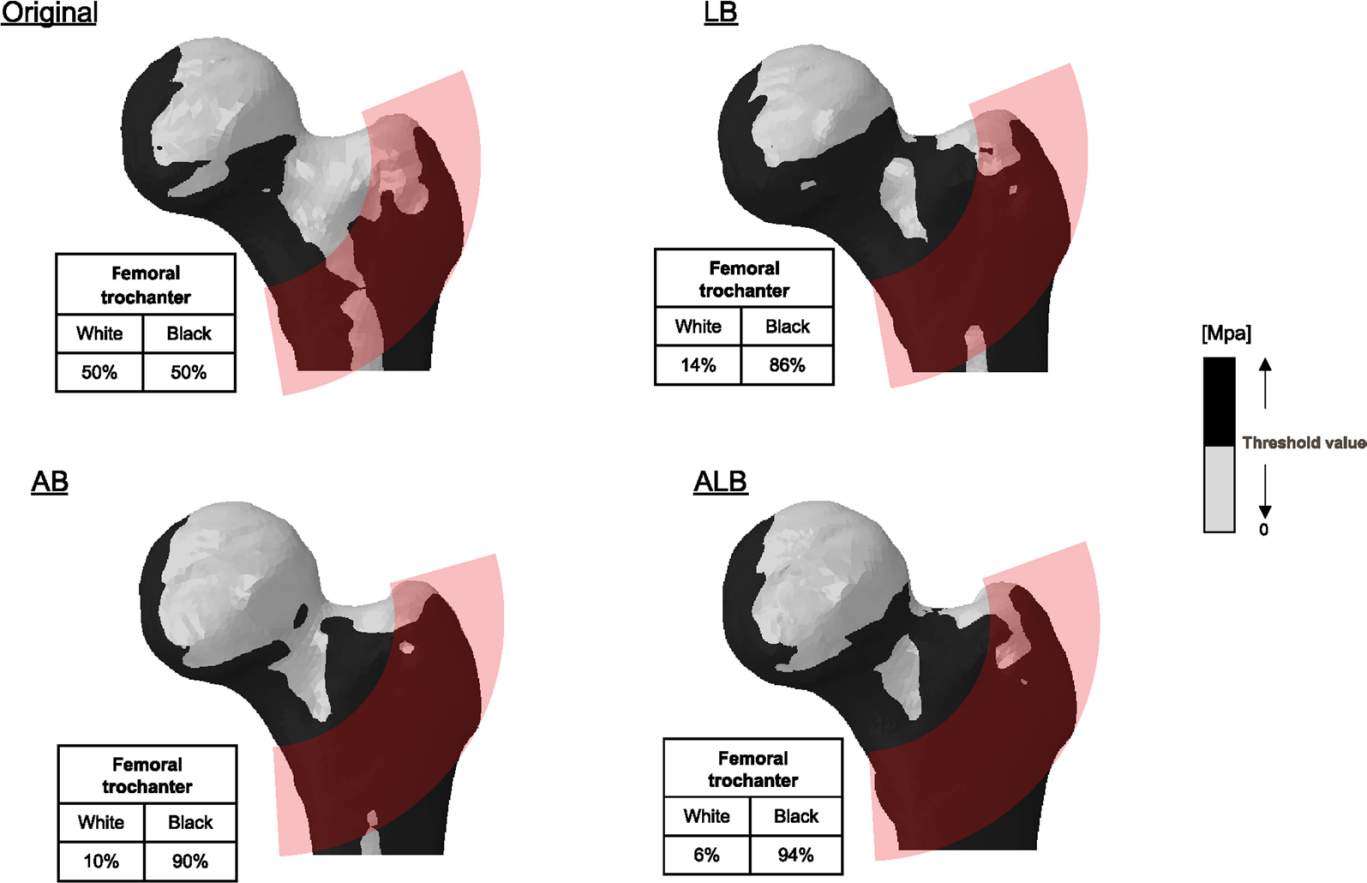
Stress distribution at the great trochanter from the anterior viewpoint: All femoral bowing indicated an expanded range of increased stress compared to the original. Among them, the black area was most expanded at 50% anterolateral bowing

**Fig. 5:**
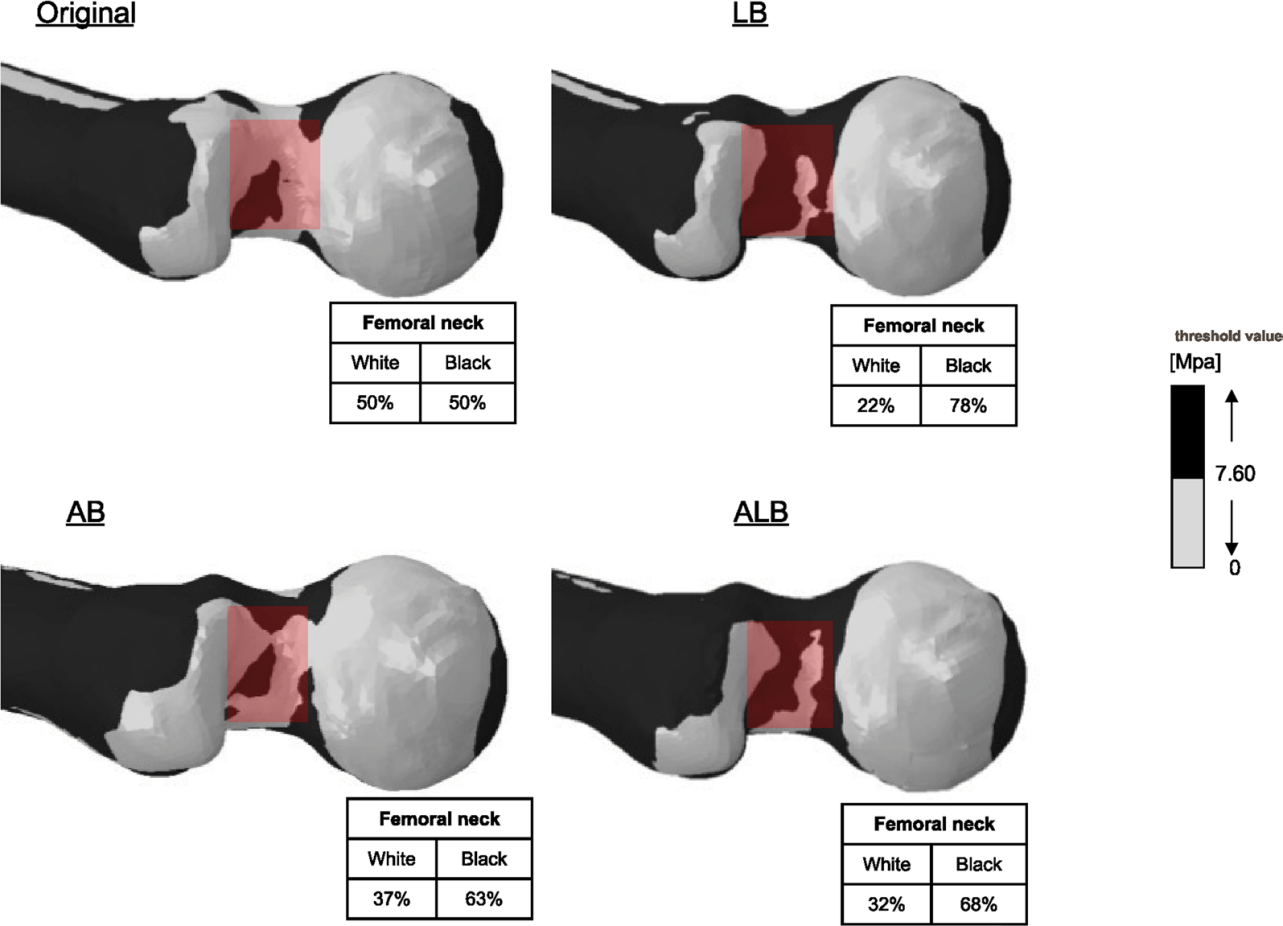
Stress distribution in the neck at the superior viewpoint: All femoral bowing showed an expanded area of increased stress compared to the original. Among these, the black area was expanded at 100% lateral bowing

In the anterior viewpoint, the stress at the neck increased for femoral bowing in both directions and was maximal at ALB (Additional file 1). The stress at the trochanter also increased, showing a maximum at ALB (Fig. 4). In the anterior viewpoint, there was more stress in the trochanter than in the neck. In the superior viewpoint, the stress at the neck increased for femoral bowing in both directions and was maximum at 1LB (Fig. 5). The stress at the trochanter also increased, with a maximum at ALB. In the superior viewpoint, the stress of ALB increased more at the trochanter than at the neck.

**Fig. 6:**
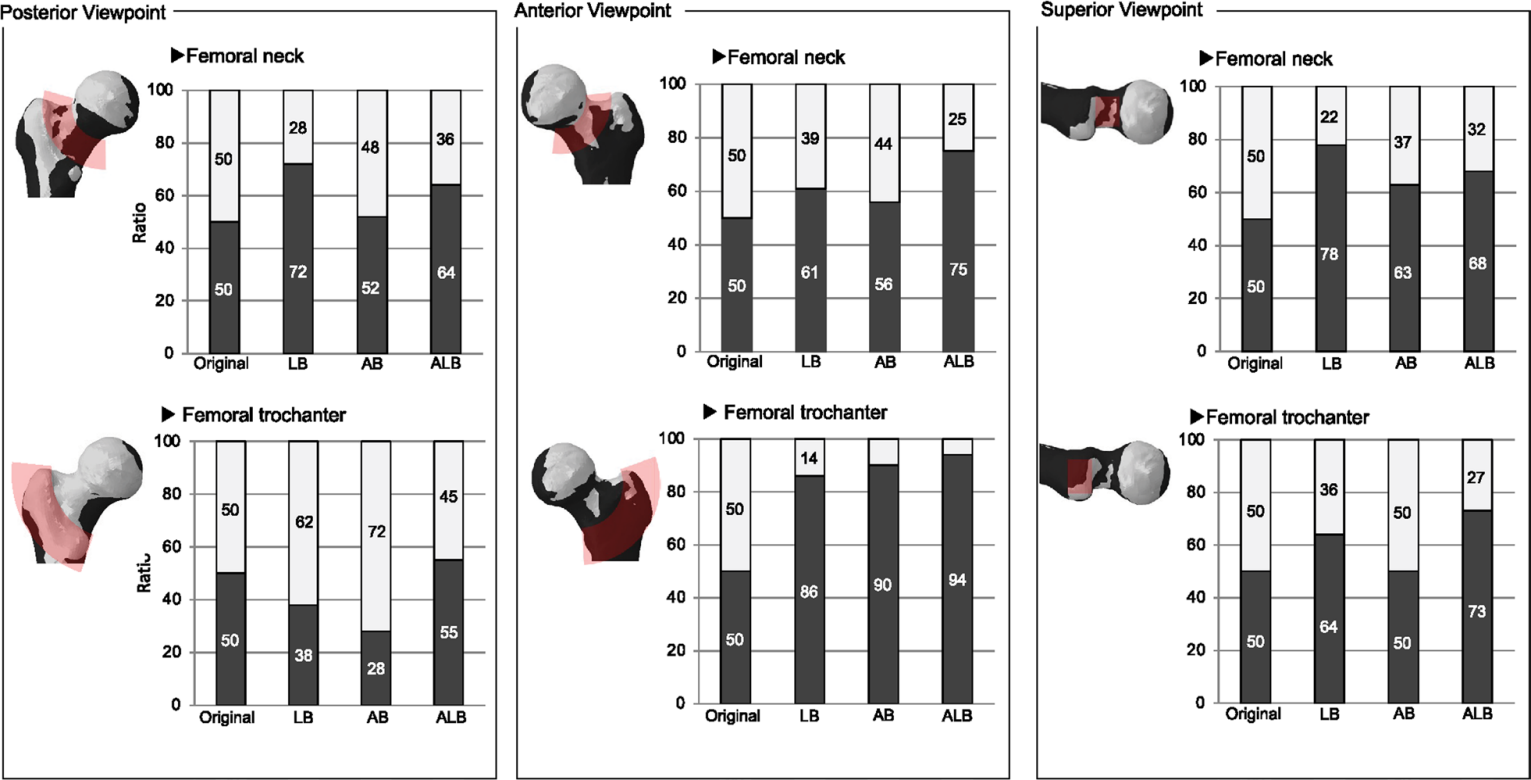
Graphical representation of the analysis results. Overall, it was found that specimens with 50% anterolateral bowing had the highest range of black areas

Across all evaluation viewpoints, stress at the neck increased in both directions of femoral bowing. In addition, the stress at the trochanter increased at ALB in all directions. In this situation, the case of the maximum ALB was the 4/6 condition. The other 2/6 conditions all had a maximum LB (Fig. 6).

**Fig. 7:**
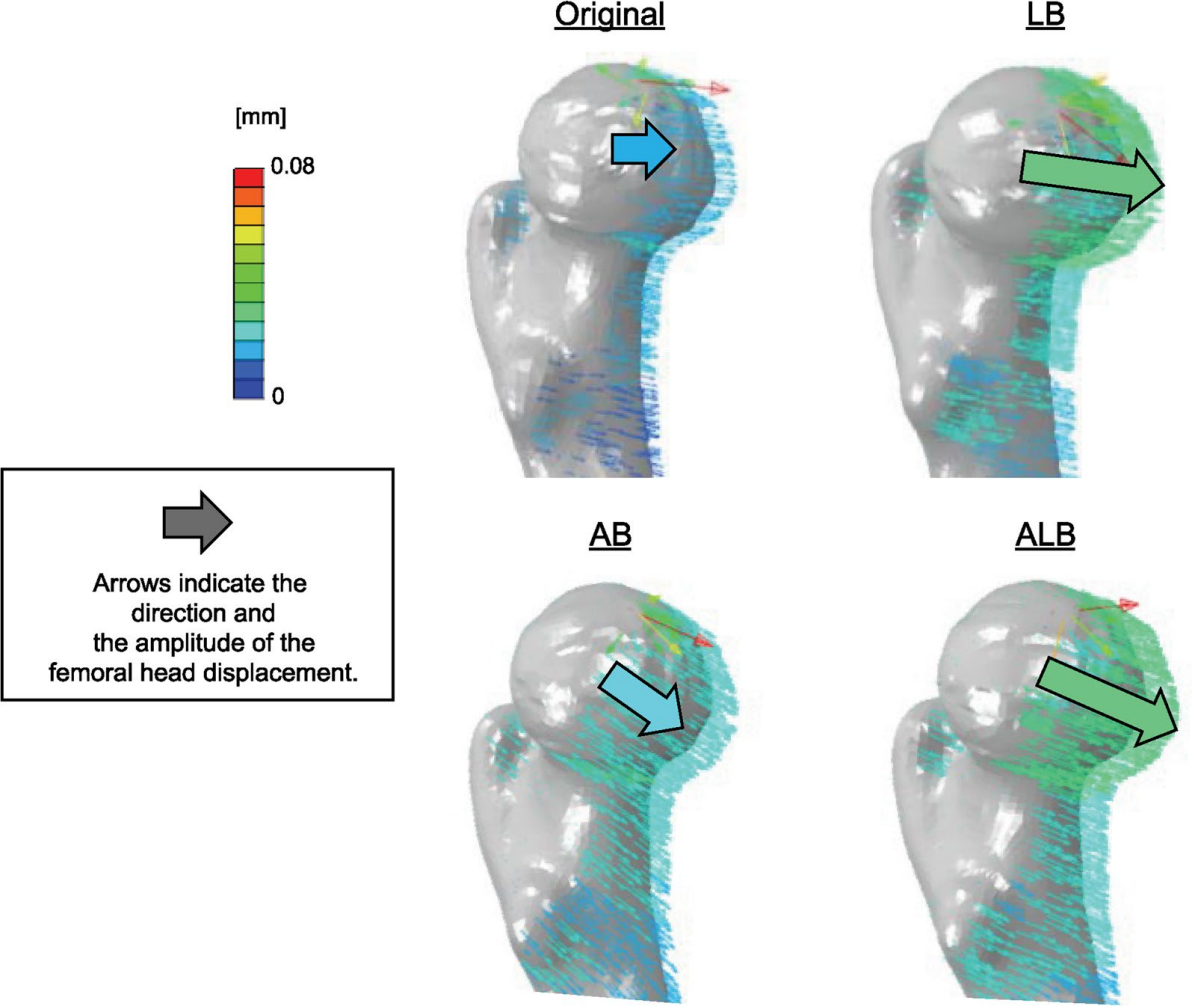
In the rotational displacement, the anteroinferior vector was more prominent in all femoral bowing models than in the original one, with a maximum at 50% anterolateral bowing. The vector direction of 50% anterolateral bowing was between 100% lateral and anterior bowing

The rotational displacement vector of AB was more significant than the original in the forward and downward directions. Similarly, that of LB was greater than AB anteriorly, and ALB had the largest vector direction between the LB and AB (Fig. 7).

## Discussion

All stresses increased in the neck due to femoral bowing, while the ALB model increased stress in all regions of interest in the trochanter. The area most susceptible to increased stress due to bowing was the anterior portion of the femoral trochanter. Four of the six regions of interest showed maximal stress in the neck and trochanter for the 5ALB model, and the other two showed maximal stress for the LB model. The anteroinferior vector of rotational displacement increased with the femoral bowing, particularly in ALB.

Most studies on femoral bowing and stress changes have been related to atypical fractures. Oh et al. performed CT-based FE analysis in patients with atypical femoral fractures (AFF). They showed that the greatest tensile stress due to loading was located adjacent to the atypical fracture site [21]. The location of damage in atypical fractures of the femur was influenced by femoral bowing and the neck-shaft angle. Similarly, Hwang et al. performed a CT-based FE analysis of patients with an atypical fracture. They reported that the femoral weakest point for tensile stress is consistent with the AFF's location. Furthermore, the AFF's location is determined by the lower extremity axis and femoral bowing [22]. As described above, FE analysis revealed that femoral bowing considerably influences atypical fractures.

Based on the results of previous studies, factors that promote femoral bowing include aging, low bone density, hypovitaminosis D, and shorter stature [15, 23]. A combination of these factors likely contributes to the occurrence of femoral bowing. These factors are also the leading causes of hip fracture. Aging, loss of muscle mass and strength, reduced sensory organ function, and decreased spatial awareness lead to easy falls [24]. The etiology of proximal femoral fractures is therefore multifactorial and specific factors may come into play in any given individual.

The results of this study demonstrated that with femoral bowing, the stresses at the femoral neck and trochanter increased, resulting in a greater rotational displacement vector to the proximal femur. The rotational displacement vectors were greater for LB and ALB than AB. Furthermore, the direction of the rotational displacement vector changed more anteroinferiorly in the ALB, suggesting that the rotational displacement of the proximal femur under load may also be greater in the ALB than in the LB (Fig. 6). This may have resulted in a higher stress value for the ALB, which is further impacted by torsion. In addition, based on previous three-dimensional evaluations [11], the femur is usually bowed anterolaterally; however, this may differ depending on the individual. This study found that the increased stress at the neck and trochanter is greater with LB than with AB; although, the clinically common ALB resulted in the most significant stress increase to the neck and trochanter. According to these FE analysis results, femoral bowing creates a stressful condition for the proximal femur, which may facilitate fracture development. It was also reported that patients aged below 75 years had more femoral neck fractures, while those over 75 years had more fractures at the trochanter [5]. The results of our study revealed that the distribution of stress in the proximal femur, where the maximum value changes from the femoral head to the femoral trochanter with increasing femoral bowing with age, may contribute to fracture lateralization.

This study has some limitations. First, CT data from only one patient was used. Second, the cortical bone thickness was configured at 1 mm uniformly. However, the thickness is non-uniform and tends to be thicker at diaphyses than at epiphyses and metaphyses. Therefore, it is important to consider the possibility that displacements may have been overestimated. Third, the bone density component was not included. Fourth, although different femoral sites in vivo have slightly different material properties of cortical and cancellous bone, the model used in this study sets them uniformly. On the other hand, the difference between our models is only bone shape because of that. Therefore, if there are differences between our results, we estimate that it may be due to bone shape. Fifth, the distal femur was fully constrained in our case. Moreover, rigid loading may lead to higher stresses than physiological constraint [25]. Therefore, it must be considered that stresses at the distal femur may be overestimated. Sixth, the values of the forces P1 and P2 in this study were set by reference to Heller et al. [19]. Sverdlova and Witzel [26] conducted a similar study with values that were set larger than ours. Finally, muscle force acting on the femur in vivo is complicated, leading to debate about more proper conditions. Seventh, only the loading conditions of walking was evaluated, but not the more severe conditions of stair climbing. Further, falls, which are a common cause of proximal femur fractures, were not simulated and evaluated. Eighth, although the rotational displacement vector was evaluated qualitatively, a quantitative evaluation was not completed. Although the degree of bowing was matched with the largest clinical case, the flex function of the software was used; therefore, there is a possibility that the bowing deformation does not correlate with actual clinical conditions.

## Conclusions

This study’s results showed that the stress distribution in the proximal femur changed with increasing femoral bowing in any direction compared to the original femoral bowing, with an increased area of higher stress in the femoral neck and trochanter compared to the original. In other words, the stress distribution in the proximal femur was lateralized due to femoral bowing. This study clarified that femoral bowing affected the stress distribution in the proximal femur. However, future studies could examine the applicability of our results to clinical practice using models and conditions that reproduce the femur more accurately. Further in-depth simulation studies are also required to clarify the mechanism of proximal femur fracture in the bowed femur, including bone density, soft tissues, muscles, and loading conditions such as walking, climbing stairs, and falling.

## Supplementary Information

**Additional file 1.** Analysis result of principal stress for checking compression tension region

## Data Availability

The datasets used and/or analysed during the current study are available from the corresponding author on reasonable request.

## Abbreviations

AB: Anterior bowing
AFF: Atypical femoral fractures
ALB: 50% Anterior and lateral bowing
CT: Computed tomography
FE: Finite element
LB: Lateral bowing
