# Supplementary material for: Influence of femoral bowing on stress distribution of the proximal femur: a three-dimensional finite element analysis
Source: J Orthop Surg Res. 2023 Feb 1;18:82. doi: 10.1186/s13018-023-03559-1 (PMC9890711; doi:10.1186/s13018-023-03559-1)
Supplement: Supplementary file 1 — Additional file 1. Analysis result of principal stress for checking compression tension region [file 13018_2023_3559_MOESM1_ESM.pdf]

Analysis Result of Principal Stress for checking compression-tension region

Stress distribution at the great trochanter from the anterior viewpoint

Color map of principal stresses with respect to the location of the tensile and compressive region

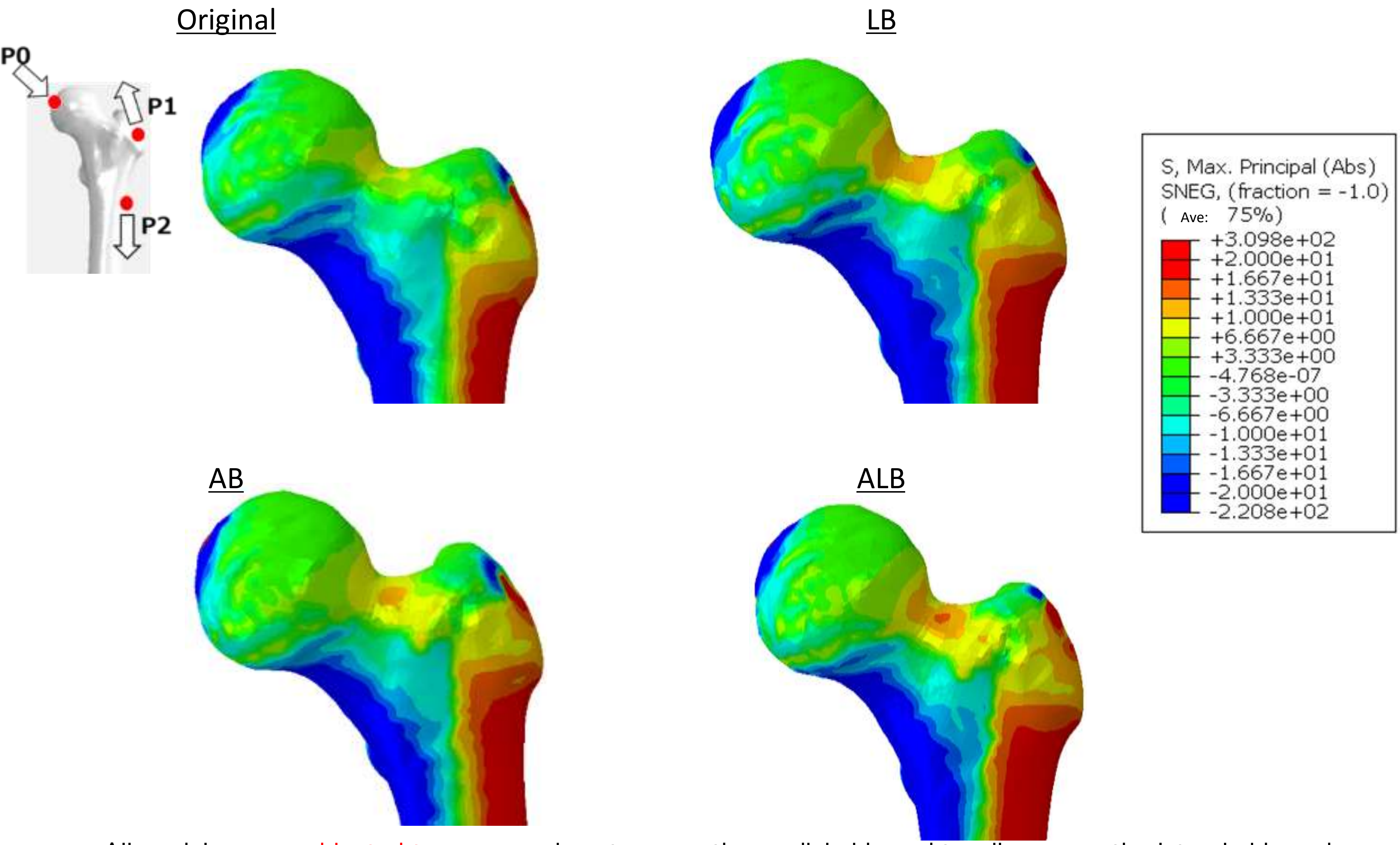

▸ All models were subjected to compressive stress on the medial side and tensile one on the lateral side and superior of the neck.

Stress distribution at the great trochanter from the posterior viewpoint

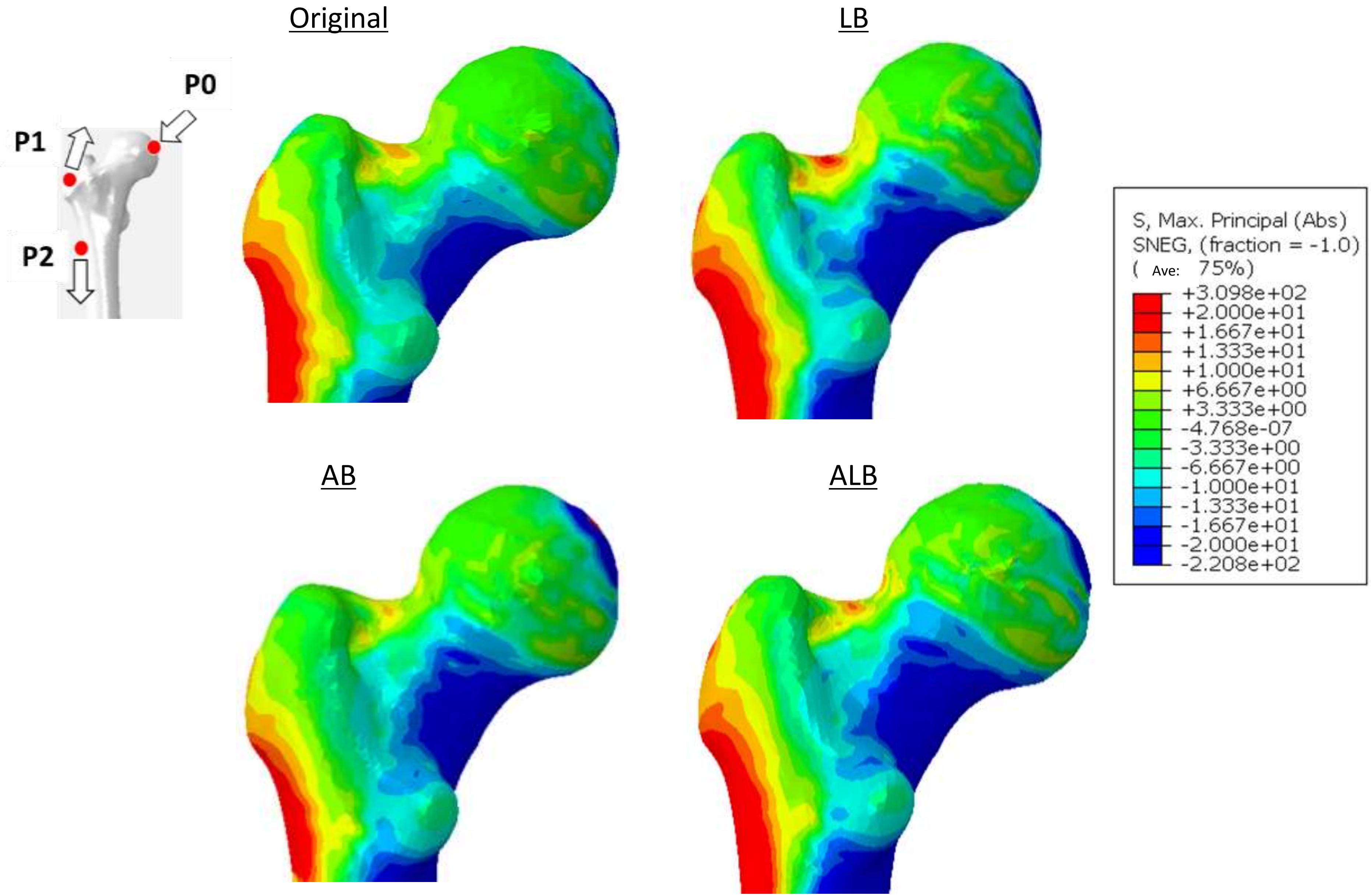

- Anteriorly, tensile and compressive regions appeared at similar locations.

Stress distribution at the great trochanter from the superior viewpoint

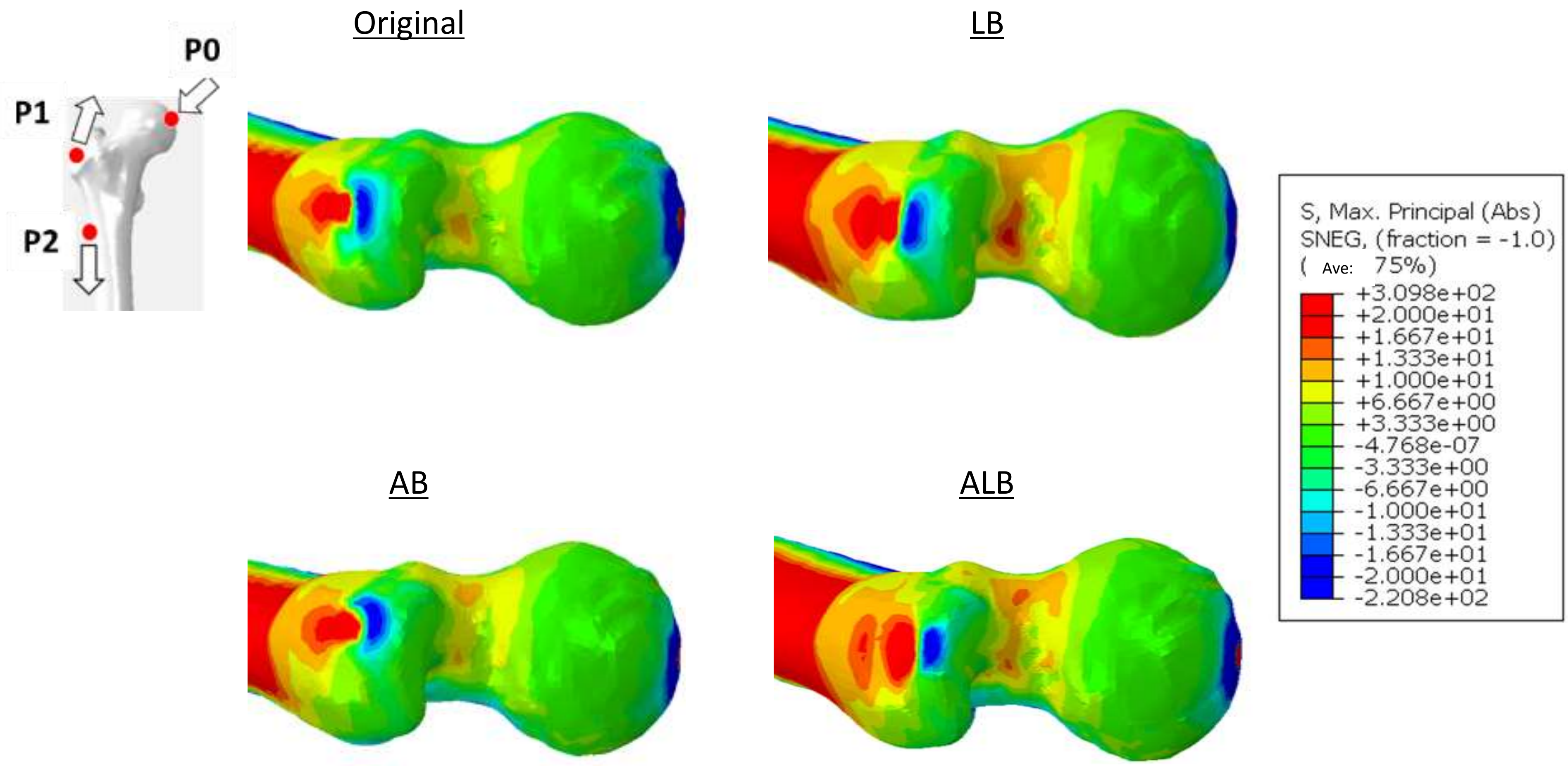

- The neck and lateral sides of all models were subjected to tensile stress
